# Stereotactic Radiosurgery for Prostate Cancer Following Magnetic Resonance Imaging Directed Biopsy: A Multidisciplinary Approach with Case Examples

**DOI:** 10.7759/cureus.2524

**Published:** 2018-04-24

**Authors:** Michael A Coker, Caleb Dulaney, Andrew McDonald, Jeffrey W Nix, Jennifer B Gordetsky, Eddy S Yang, Michael C Dobelbower, Soroush Rais-Bahrami

**Affiliations:** 1 School of Medicine, University of Alabama at Birmingham; 2 Department of Radiation Oncology, University of Alabama at Birmingham; 3 Department of Urology, University of Alabama at Birmingham; 4 Department of Pathology, University of Alabama at Birmingham

**Keywords:** multiparametric mri, stereotactic body radiotherapy

## Abstract

Classically, prostate cancer has been diagnosed via systematic, transrectal ultrasound-guided biopsy prompted by an abnormal digital rectal exam or elevated serum prostate-specific antigen (PSA) level. The development of multi-parametric magnetic resonance imaging (MRI) has led to improved detection of prostate cancer foci. For patients with clinically localized prostate cancer seeking definitive therapy through radiation therapy, external beam radiation has been a mainstay with a movement toward hypofractionation, notably prostate stereotactic body radiotherapy (SBRT). We aim to describe the practical aspects of establishing a multidisciplinary, MRI-based prostate SBRT program by means of case examples.

The prostate SBRT team at the University of Alabama at Birmingham has been performing prostate SBRT for over four years using a multidisciplinary workflow. We have additionally completed a phase II trial of prostate SBRT with additional targeting of intraprostatic lesions with higher doses of radiation using a simultaneous integrated boost technique.

While there have been no reported randomized trials of prostate SBRT, this treatment has been proven safe and effective for properly selected patients with low and intermediate-risk prostate cancer. We present our multidisciplinary approach to prostate SBRT with two clinical cases targeting high-risk [MAM1] lesions in different anatomic zones of the prostate highlighting pertinent clinical challenges in successfully delivering prostate SBRT and managing potential side effects.

In conclusion, we report a multidisciplinary, MRI-based approach to treating patients with ultra hyperfractionated stereotactic radiosurgery as primary definitive treatment for prostate cancer.

## Introduction

Prostate cancer is the most common non-cutaneous cancer diagnosed in American men. An estimated 180,890 patients will be diagnosed annually resulting in approximately 9% of all cancer-related deaths [1]. Classically, prostate cancer has been diagnosed via systematic sampling of the prostate gland with transrectal ultrasound (TRUS)-guided biopsy prompted by an abnormal digital rectal exam or elevated serum prostate-specific antigen (PSA) level.

The development of advanced, multi-parametric magnetic resonance imaging (MP-MRI) has led to improved detection of prostate cancer foci and allows for targeting areas of suspicion for needle biopsy [2]. Multiple centers have shown increased detection of clinically significant prostate cancers based on targeted biopsy over the standard, systematic biopsy approach [3, 4, 5].

For patients with clinically localized prostate cancer seeking definitive therapy with curative intent, radical prostatectomy and radiation therapy have been the primary treatment options. Of the radiation therapy approaches available, external beam radiation therapy has been a mainstay in the treatment of prostate cancer for decades. However, the protracted course, often consisting of greater than 40 treatment sessions lasting as long as nine weeks, is unfavorable to some patients and clinicians. Prostate stereotactic body radiotherapy (SBRT) can be performed with multiple radiation therapy platforms including the widely available linear accelerator (LINAC), helical tomotherapy, and robotic radiosurgery. Overall, biochemical control and early toxicity with prostate SBRT appear similar to both standard fractionation and moderately hypofractionated external beam radiation regimens [6].

The convenience of prostate SBRT and appeal to an expanded range of patients has made it a favorable alternative to more protracted courses of radiation therapy despite a lack of randomized evidence. A multidisciplinary approach is necessary to ensure patients receive safe and effective prostate SBRT. In addition, the use of MRI encourages multidisciplinary collaboration and enhances the efficacy of targeted biopsy and therapy. The purpose of this paper is to describe the practical aspects of establishing a multidisciplinary, MRI-based prostate SBRT program and areas where urologists can provide particular expertise in the radiation planning process.

## Case presentation

Methods

The prostate SBRT team at the University of Alabama at Birmingham has been performing prostate SBRT for over four years using a multidisciplinary workflow. We have additionally completed a phase II trial (NCT01856855) of prostate SBRT with additional targeting of intermediate to high-risk intra-prostatic lesions with higher doses of radiation using a technique called simultaneous integrated boost (SIB). In this study, we describe our multidisciplinary approach to delivering safe, high-quality prostate SBRT, and present our experience by highlighting representative cases.

MP-MRI protocol and MRI-targeted biopsy

The majority of patients who underwent stereotactic radiosurgery for prostate cancer treatment at our institution had prostate cancer diagnosed on MRI/TRUS fusion-guided prostate biopsy. These patients underwent multi-parametric MRI of the prostate utilizing a 3.0 Tesla MRI and a phased-array surface coil as previously described [7].

The multiparametric MRI studies were reviewed at a multidisciplinary prostate imaging conference attended by fellowship-trained body radiologists and urologic oncologists trained in prostate MRI and MRI/TRUS fusion-guided biopsy procedures, respectively. In the setting of this multidisciplinary imaging conference, consensus interpretation was reached for each case identifying lesions within the prostate concerning for harboring prostate cancer. Additionally, using the DynaCAD (InVivo Corp, Gainesville, FL) post-image processing software, whole gland three-dimensional prostate volumes and internal three-dimensional lesions of suspicion for harboring prostate cancer were segmented during the imaging conference. Patients with lesions suspicious for malignancy were offered MRI/TRUS fusion biopsy in addition to standard systematic random TRUS biopsy. MRI/TRUS fusion biopsy was performed using the UroNav system (Philips/InVivo, Gainesville, FL, USA). At least two needle cores were sampled from different directions for each three-dimensionally segmented lesion of suspicion as recommended for optimized targeted sampling [8].

Patient selection and treatment counseling

Potential patients for prostate SBRT have Karnofsky performance status >60, life expectancy >5 years, and histologically confirmed prostate adenocarcinoma with very low, low, or intermediate NCCN risk stratification based on clinical T stage (≤T2a), PSA (<20 ng/ml), and Gleason score (≤7) [9]. Particular risk factors for toxicity and reasons to favor conventional radiation include a history of inflammatory bowel disease, previous prostate or urethral procedures, ongoing immunosuppressive therapy, ongoing antiplatelet therapy, and prostate volume over 120 cc.

Fiducial marker placement

The amelioration of image-guided radiotherapy with fiducial placement has seen improved outcomes for men with prostate cancer (PCa) treated with hypofractionated stereotactic radiosurgery. All ongoing randomized trials require fiducial markers, as these are an important aspect of insuring accurate delivery. In addition, fiducial markers can be monitored in real-time during treatment.

Markers are placed in a triangular arrangement without a necessary association to the imaged and tissue-proven areas of intraprostatic cancer foci. The urethra should be avoided in order to minimize the loss of markers through erosion into the urethral channel and voiding them with urine. “Tenting” of the prostate by the needle, the appearance of the needle being inside the prostate on TRUS when it has not actually penetrated the prostate capsule, may explain further loss of some markers in the space between the prostate and rectum [10].

RT simulation and planning

Radiation treatment planning is done using computed tomography (CT) scan of the pelvis with the patient in supine position with full bladder and empty rectum. Full bladder is preferred for treatment and therefore is necessary during the treatment planning process. Full bladder lowers the average radiation dose to the bladder and reduces toxicity. The bladder should be filled reproducibly, i.e., not over-filled to the point of discomfort or incontinence. This can be accomplished by drinking 16-24 ounces of fluid 1 hour prior to simulation and treatment. Urinary catheters are not generally used in ongoing clinical trials for treatment or simulation. Delineation of the prostatic urethra can be challenging even with MRI. While a urinary catheter could make delineation easier, we feel it could cause distortion of the prostate and is not reproducible during treatment unless the catheter is used for all treatments, which may be unnecessarily invasive.

MRI images are fused with the CT, and the radiation oncologist and urologist identify the prostate target volume and the dominant intraprostatic lesion. Radiation oncologists delineate the volume that will be targeted with therapy and adjacent organs that will be avoided. The target is the entire prostate while the bladder, rectum, urethra, femoral heads, and small bowel are generally considered organs at risk. Urologists can be particularly helpful in the target delineation process by helping identify the prostate capsule, the neurovascular bundles, the urethra, and the prostate apex as these anatomic landmarks and boundaries are commonly evaluated on pre-treatment MRI by urologists planning surgical treatment for prostate cancer patients. Institutional radiation planning goals for the rectum, bladder, and urethra are outlined in Table 1. Planning goals for the prostate and high-risk target volume are outlined in Table 2. The entire prostate target is prescribed a dose of 36.25 Gy in five treatments while the smaller high-risk target volume is simultaneously prescribed a dose of 40 Gy in the same five treatments.

**Table 1:** Radiation therapy planning goals for normal tissues. Presented are representative planning goals used for prostate stereotactic body radiotherapy (SBRT). Each goal represents the maximum acceptable volume of each organ that should receive the specified dose. The percent dose represents the percent of the prescribed dose to the entire prostate (36.25 Gy). Meeting these planning goals often takes priority over complete coverage of the high-risk target volume (40 Gy).

Organ	Volume	Dose (Gray)
Rectum	Maximum point dose	38.06 (105% of 36.25 Gy)
	<5%	36.25 (100%)
	<10%	32.63 (90%)
	<20%	29.0 (90%)
	<50%	18.13 (50%)
Bladder	Maximum point dose	38.06 (105%)
	<10%	32.63 (90%)
	<50%	18.13 (50%)
Urethra	Maximum point dose	38.79 (107%)

**Table 2:** Radiation therapy planning goals for target volumes. Presented are representative planning goals for prostate stereotactic body radiotherapy (SBRT). Two target volumes are used in the planning process. The entire prostate plus a margin for motion and setup error receives 36.25 Gy. The high-risk target lesion plus a margin for motion and setup error receives 40 Gy. Each planning goal represents the minimum volume that should receive the specified dose. Coverage of these targets generally must respect normal tissue planning goals.

Target	Volume	Dose (Gray)
Prostate	95%	36.25 (100% of 36.25 Gy, ideal)
	95%	34.4 (95%, acceptable)
	Minimum point dose	34.4 (95%)
High-risk lesion	95%	38 (95% of 40 Gy)

RT delivery

Patients are initially aligned by orthogonal kilovoltage X-rays and then a cone-beam CT scan is performed using on-board imaging technology. The urologist and radiation oncologist are present and evaluate patient positioning based on fiducial alignment and alignment of the prostate-rectum interface on kV imaging and cone-beam CT imaging at the time of actual SBRT treatment. We generally treat patients on five non-consecutive days with a goal to complete therapy within two weeks.

Case presentations

Below, we present our multidisciplinary approach to prostate SBRT with two cases targeting high-risk lesions in different regional zones of the prostate and highlighting pertinent clinical challenges in successfully delivering prostate SBRT and managing potential side effects.

Case 1

A 64-year-old African-American male presented with an elevated PSA of 9.3 ng/mL and no previous history of prostate biopsy. He had a systematic TRUS-guided extended sextant biopsy with two of 12 cores demonstrating prostate cancer, one with GS 4+3 and a second with GS 3+4, both in the left apical region. He had no baseline urinary or bowel problems, but did have erectile dysfunction adequately managed with sildenafil taken as needed for sexual performance. His AUA urinary symptom score was 3 and SHIM score was 14 without use of PDE5 inhibitors. Using the web-based Memorial Sloan Kettering Cancer Center nomogram, his risk of nodal involvement was estimated to be 7% [11]. He was in good overall health and his age-adjusted life expectancy was estimated to be 19.4 additional years using the Social Security Administration life tables. After discussion of all treatment options with the multidisciplinary team, he elected to pursue definitive treatment with prostate SBRT.

**Figure 1:**
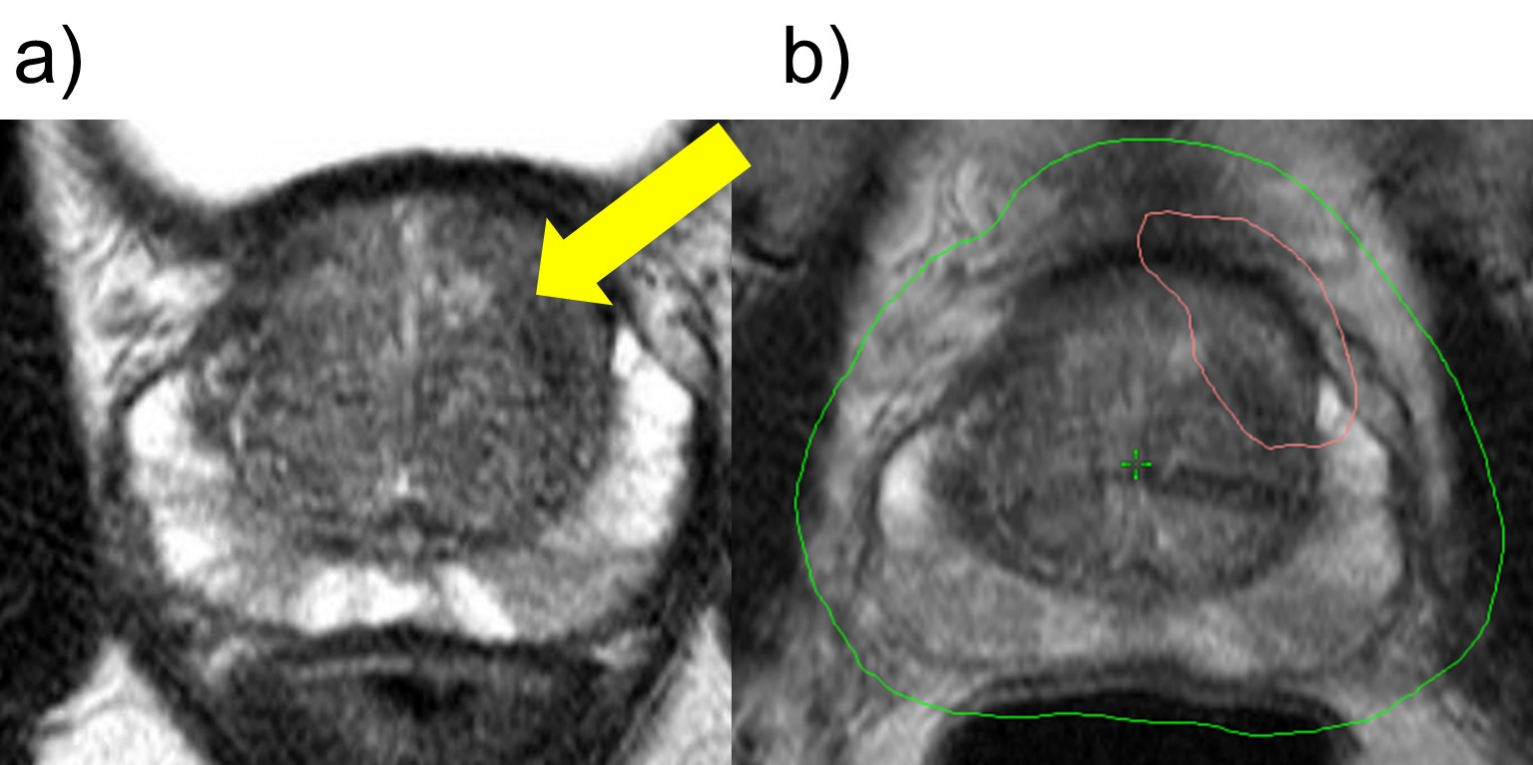
Case 1 shows a left anterior transition zone lesion treated with stereotactic body radiotherapy. (a) Hypointense left anterior transition zone lesion with suspicious PI-RADS 5 score radiographic features (indicated by arrow) on T2 magnetic resonance imaging (MRI). (b) Green and red distributions indicate 36.25 Gy and 40 Gy radiation isodose lines, respectively.

Diagnostic multi-parametric prostate MRI and review by the multidisciplinary prostate imaging conference previously demonstrated a 1.7 cm T2-weighted hypointense lesion with corresponding restricted diffusion in the anterior apical transition zone left of midline that was considered high suspicion based on imaging parameters but associated well to the systematic biopsy cores positive for prostate cancer (Figure 1). The whole prostate volume and the area of MRI cancer suspicion were segmented using post image processing software. Following the MRI, three gold fiducial markers were placed in the urology office via a TRUS-guided approach. The radiation therapy planning CT scan was scheduled two weeks after fiducial placement. No urinary catheter, rectal balloon, or rectal spacer was used.

After the simulation CT scan was completed, axial T2-weighted and post contrast T1-weighted MRI were fused with the CT images using radiation therapy planning software. Target volumes of the prostate and the high-risk lesion were then generated on the CT based on MRI segmented regions of suspicion. The radiation oncologist and urologist met to review the image fusion and target volumes prior to proceeding with final treatment planning. In this case, the highest suspicion lesion was located in the left anterior apical prostate as visible on T2-weighted MRI. Hence the prostate surrounding the high-risk lesion was included in the high-risk target volume for planned SIB (Figure 1). For transition zone and central gland lesions, limiting dose to the urethra presents the greatest challenge to radiation therapy planning. However, most prostate SBRT protocols recommend achieving normal tissue dose limits over complete coverage of the high-risk target volume. In this case, adequate coverage was possible while limiting urethral dose to an acceptable range.

The high-risk volume received 40 Gy SIB in five fractions while the entire prostate received 36.25 Gy. He was placed on tamsulosin during radiation therapy after developing dysuria, diminished urinary stream, and frequency corresponding to an increased AUA symptom score of 14. These irritative urinary symptoms were his only radiation-associated side effects. He did not develop any significant lower gastrointestinal symptoms. Six months after his treatment, he continued to have irritative urinary symptoms and remained on tamsulosin.

Case 2

A 65-year-old Caucasian male presented with an elevated PSA of 8.98 ng/mL and a history of TRUS-guided extended sextant biopsy negative for prostate cancer three years prior to presentation. At the time of his prior prostate biopsy, his PSA was 7.25 ng/mL. He had mild baseline lower urinary tract symptoms with an associated AUA urinary symptom score of 14. He denied any erectile dysfunction and had a SHIM score of 25. MP-MRI was performed, and review by the multidisciplinary prostate imaging conference revealed patchy diffuse abnormal signal that is often seen in patients with prior biopsy history and/or inflammation. Despite this diffuse irregularity in signal in the right posterolateral peripheral zone, there was a focal area of well-defined hypointensity with corresponding diffusion restriction suspicious for harboring prostate cancer. There was notable central gland hyperplastic nodules with regional areas of low T2 signal intensity in the left anterior transition zone that was low suspicion for representing malignancy more likely representing benign prostatic hyperplasia nodularity. With these findings, he underwent MRI/TRUS fusion-targeted biopsy that demonstrated GS 4+3 adenocarcinoma in 80% of the specimen cores sampled from the MRI-targeted lesion in the posterior peripheral zone. After discussion of all treatment options with the multidisciplinary team, he elected to proceed with prostate SBRT.

**Figure 2:**
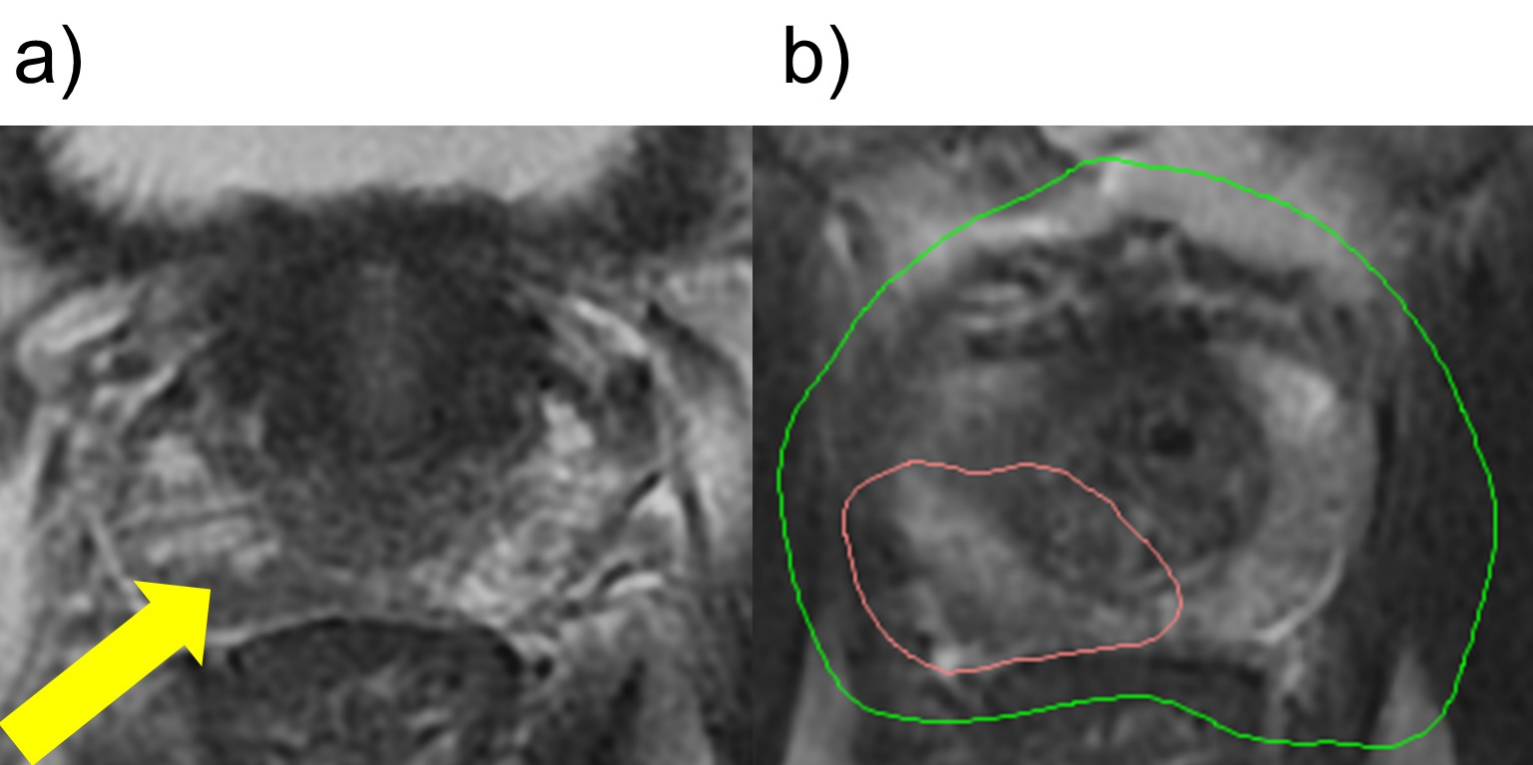
Case 2 shows a right posteriolateral peripheral zone lesion treated with stereotactic body radiotherapy. (a) Hypointense, right posteriolateral peripheral zone lesion with suspicious radiographic features (indicated by arrow). (b) Green and red distributions indicate 36.25 Gy and 40 Gy radiation isodose lines, respectively.

The patient then had TRUS-guided fiducial markers placed followed by the radiation therapy planning CT scan two weeks after fiducial marker insertion. CT and MRI fusion was performed to allow for CT generation of the target volumes of the prostate gland and the high-risk intraprostatic lesion which was used for MRI-targeted biopsy proven to represent the intermediate-risk prostate cancer. The radiation oncologist and urologist met to review this image fusion between treatment simulation CT and MP-MRI as well as target volumes for SBRT and SIB. In this case, the highest suspicion lesion was located in the posterior peripheral zone, well visualized on T2-weighted MRI around which the SIB region was drawn. Dose limitations in the region of the rectum were considered posteriorly, and the ipsilateral neurovascular bundle exposures were considered but not considered dose limiting (Figure 2).

The SIB volume in the posterior peripheral zone received 40 Gy in five fractions while the entire prostate gland was administered 36.25 Gy over the same time course. The patient was being managed with tamsulosin prior to starting radiation therapy due to his history of lower urinary tract symptoms. He developed moderate dysuria during the final two fractions of radiation therapy that was managed by increasing his tamsulosin to twice daily dosing. One week after radiation was completed, he developed a self-limited diarrhea lasting three days. By his one-month follow-up visit, his urinary and gastrointestinal symptoms had returned to baseline.

## Discussion

The utilization of prostate stereotactic radiotherapy has been widely adapted as a mainstay in the management of prostate cancer, as evidenced by an increase in application from 0.1% to 3.9% for all patients treated with radiation [12]. Multiple prospective trials have shown biochemical recurrence-free survival rates of 90-100% for low-risk, and 84-100% for intermediate-risk prostate cancer [13].

Katz et al. enrolled 304 low, intermediate, and high-risk patients to undergo treatment with five fractions of either 7 or 7.25 Gray. There were no incidents of acute grade 3 or 4 toxicity, and only one late grade 3 toxicity at the 12-month follow-up [14]. Anwar et al. constructed upon the foundation of the Katz study, expanding the use of stereotactic radiotherapy to include intermediate- and high-risk prostate cancer. Of a total of 50 patients treated with 9.5 or 10.5 Gy, the biochemical recurrence-free survival was 83%, and no grade 3 or higher toxicity was noted [15]. The American Society for Radiation Oncology (ASTRO) released a policy statement in 2013 in support of the utilization of stereotactic radiotherapy as a substitute for traditional radiotherapy in low- to intermediate-risk disease. Ongoing trials, such as the SMART trial at Duke University, a prospective phase II study for stage T1-T2c prostate cancer, continue to seek the answer to the efficacy and toxicity of prostate stereotactic radiotherapy.

While there have been no reported randomized trials of prostate SBRT, this treatment appears to be a safe and effective form of therapy for properly selected patients with low and intermediate-risk prostate cancer. Ideally, prostate SBRT should be done in a multidisciplinary setting by a team where urologists and radiation oncologists work hand-in-hand to identify proper candidates and ensure that high suspicion intra-prostatic lesions are adequately treated with radiation therapy. Prostate MRI is particularly effective in this setting as it allows for targeted biopsy and confirmation of high-risk lesions that can be targeted with escalated radiation dose.

## Conclusions

In conclusion, we report a multidisciplinary, MRI-based approach to treating patients with ultra hyperfractionated stereotactic radiosurgery as primary definitive treatment for prostate cancer. Our center has found this multidisciplinary approach optimizes patient selection, counseling, image processing, and treatment workflow with the goal of improving patient experiences and outcomes. Further studies are underway to investigate intermediate long-term outcomes in patients treated through this multidisciplinary algorithm.
